# Ecomorphological diversity of Australian tadpoles

**DOI:** 10.1002/ece3.4733

**Published:** 2018-11-26

**Authors:** Emma Sherratt, Marion Anstis, J. Scott Keogh

**Affiliations:** ^1^ Department of Ecology and Evolutionary Biology, School of Biological Sciences The University of Adelaide Adelaide South Australia Australia; ^2^ School of Environmental and Life Sciences The University of Newcastle Callaghan New South Wales Australia; ^3^ Australian Museum Research Institute Australian Museum Sydney New South Wales Australia; ^4^ Division of Ecology & Evolution, Research School of Biology The Australian National University Canberra Australian Capital Territory Australia

**Keywords:** anatomy, anura, ecology, macroevolution, morphology

## Abstract

Ecomorphology is the association between an organism's morphology and its ecology. Larval anuran amphibians (tadpoles) are classified into distinct ecomorphological guilds based upon morphological features and observations of their ecology. The extent to which guilds comprise distinct morphologies resulting from convergent evolution, the degree of morphological variability within each guild, and the degree of continuity in shape between guilds has not previously been examined in a phylogenetically informed statistical framework. Here, we examine tadpole ecomorphological guilds at a macroevolutionary scale by examining morphological diversity across the Australian continent. We use ecological data to classify species to guilds, and geometric morphometrics to quantify body shape in the tadpoles of 188 species, 77% of Australian frog diversity. We find that the ecomorphological guilds represented by Australian species are morphologically distinct, but there is substantial morphological variation associated with each guild, and all guilds together form a morphological continuum. However, in a phylogenetic comparative context, there is no significant difference in body shape among guilds. We also relate the morphological diversity of the Australian assemblage of tadpoles to a global sample and demonstrate that ecomorphological diversity of Australian tadpoles is limited with respect to worldwide species. Our results demonstrate that general patterns of ecomorphological variation are upheld in Australian tadpoles, but tadpole body shape is more variable and possibly generalist than generally appreciated.

## 1 INTRODUCTION

The association between an organism's morphology and its ecology, known as ecomorphology (Karr & James, 1975; Williams, 1972), is a pervasive concept in biology. The two are naturally linked because ecological factors often play a strong selective role on morphological variation (e.g., Muschick, Barluenga, Salzburger, & Meyer, 2011, Anderson, Renaud, & Rayfield, 2014), and morphology typically determines the performance of an organism in its environment (e.g., Stayton, 2011, Anderson & Patek, 2015). Particularly apparent in fish‐like body forms, the clear functional relationship between body shape and swimming performance is governed by hydrodynamic effects which in turn are related to the aquatic environments in which the animals live (Webb, 1984, reviewed in Blake, 2004, Lauder, 2015). The fish‐like body shape of tadpoles also reflects ecological niches and locomotive strategies. Tadpoles are the free‐living aquatic larval stage of frogs and toads, with a distinctive bauplan, comprising a composite head and body, and a muscular tail (Altig & McDiarmid, 1999a). Their aquatic locomotion is awkward compared to fish (Liu, Wassersug, & Kawachi, 1997; Wassersug & Hoff, 1985), however hydrodynamic studies suggest that this is an adaptation to maintain locomotive efficiency while developing lateral‐jutting hindlimbs during metamorphosis (Liu et al., 1997; Liu, Wassersug, & Kawachi, 1996).

The concept of “ecomorphological guilds” is based upon this functional relationship between body shape and ecology and exists to act as a proxy for studying assemblages of aquatic environments and modeling ecosystems (e.g., Bower & Piller, 2015, Oliveira et al., 2010, Balon, 1975). The ecomorphological guilds of tadpoles were developed and classified as a result of the recognition that tadpoles inhabiting particular ecological niches display distinct, potentially convergent, forms (Altig & Johnston, 1989; Altig & McDiarmid, 1999b; Orton, 1953; Van Dijk, 1972). Orton (1953) defined four tadpole morphotypes based on the keratinized mouth parts of the oral disc (subsequently recovered as statistically distinct by Roelants, Haas, and Bossuyt (2011)). Orton was the first to visually present the “adaptive radiation in tadpoles,” illustrating seven different types that relate to diverse ecologies, and postulated on convergent evolution in tadpoles of fast‐flowing streams, and in surface‐feeding tadpoles, based upon qualitatively similar external morphology. Altig and Johnston (1989) built upon this and proposed a formal qualitative classification framework of tadpole ecomorphological guilds, which since has been the standard for taxonomic and fossil tadpole descriptions (e.g., McNamara et al., 2010). The primary morphological traits pertaining to ecomorphological variation in tadpoles are oral disc (mouth structures) position and tail shape and microhabitat use. For example, the suspension‐feeding guild has a dorsally positioned oral disc, small tail fins and inhabits the water column; the nektonic guild has large, well‐arched tail fins for swimming freely in open water and an anteroventral oral disc. Tadpole external morphology, usually examined in the lateral view, is thus considered to confer ecomorphological guilds. However, since this framework was proposed, the extent to which guilds comprise distinct morphologies resulting from convergent evolution, the degree of morphological variability within each guild, and the degree of continuity in shape between guilds has not been examined in a phylogenetically informed statistical framework (but see Marques & Nomura, 2015).

Here, we investigate tadpole ecomorphological guilds at a macroevolutionary scale in a phylogenetic context. We use a continent‐wide sample of species and characterize the body shape diversity of Australian tadpoles using geometric morphometrics. Our sample consists of 77% of the total native amphibian diversity in Australia, comprising two Neobatrachian families that have a free‐living larval stage: Hylidae and Myobatrachidae. We exclude all microhylid species and four myobatrachid species because these species are direct developers with no free‐living tadpole stage. We then classify these taxa by their ecomorphological guild based on ecological and behavioral information presented in Anstis (2013). We test whether there are guild‐specific body shape differences and assess the morphological variation within each type, and show that because of shared features of the microenvironment, ecomorphological guilds in fact form a continuum in morphospace. Finally, we compare the observed diversity to that of non‐Australian species that exemplify the ecomorphological guilds proposed by Altig and Johnston (1989), and demonstrate that ecomorphological diversity of Australian tadpoles is limited with respect to worldwide species. This is the first study to comprehensively examine ecomorphology in larval anurans at the continental scale.

## 2 MATERIALS AND METHODS

Our sampling is extensive from the continent of Australia, which is home to 242 native anuran species (AmphibiaWeb), excluding the recent invasive migrant, the cane toad (*Bufo marinus*) and the migrant *Papurana daemeli* (Ranidae; formerly *Hylarana*). In total, we sampled 187 species with a distinct larval stage (Supporting information Table S1, details of these are given below) from the precise drawings of preserved specimens in the comprehensive work of Anstis (2013), which have been used in previous studies (Sherratt, Vidal‐García, Anstis, & Keogh, 2017; van Buskirk, 2009). The sampled tadpoles are at a similar stage in their development (mean Gosner stage 35.4, ±3.09) when the hindlimb bud is visible with very short toe nubs. We excluded the 28 direct developing species from this study because they do not progress through a tadpole‐like body shape larval stage, and we could not include the pouch‐brooding frog (*Assa darlingtoni*) in this study because the tadpole illustrated in Anstis (2013) is significantly more developed than Gosner stage 35.

Ecomorphological guilds of larval amphibians outlined by Altig and Johnston (1989) remain the most comprehensive guide to date. They defined 24 ecomorphological guilds (not including sub‐types), divided into two groups based on trophic mode, endotrophic and exotrophic, under which each category there is a nested classification system (explained below). Our data herein use the tadpole ecology and behavior for most Australian species detailed in Anstis (2013) (with additional information where possible from descriptions in Altig and McDiarmid (1999b)) and applies the definitions in Altig and Johnston (1989) to classify all species to an ecomorphological guild.

Endotrophic species obtain their entire developmental energy from yolk (Thibaudeau & Altig, 1999) and comprise six guilds, of which three are relevant here: “paraviviparous” —froglet hatches from egg at site of deposition, intimately associated with parent's body; “direct developers”—froglet hatches from egg at site of deposition, not intimately associated with parent's body; and “nidicolous”—free‐living, non‐feeding tadpole remains in nest until metamorphosis. Note that tadpole is the common term for the free‐living larval stage of anurans, but it specifically refers to non‐reproductive larvae that are endotrophic‐nidicolous or exotrophic (McDiarmid & Altig, 1999). Herein, we use tadpole for brevity, because all but one species under study fall under this definition; the exception is paraviviparous gastric brooding frog *Rheobatrachus silus, *which has a relatively tadpole‐like body plan and is included for phylogenetic completeness. Of the endotrophic species we sampled (*N* = 9), most are “nidicolous,” either developing in a viscous jelly within a terrestrial nest (*Geocrinia *and *Philoria loveridgei*), or in small water‐filled cavities in terrestrial burrows (remaining *Philoria*), and one is “paraviviparous,” developing in the stomach of the mother (*Rheobatrachus silus*).

Exotrophic tadpoles, on the other hand, feed actively while developing in an aquatic environment, and comprise the remaining 18 guilds, divided up into two groups according to water source (lotic and lentic). While tadpoles generally require a freshwater source in which to develop. there is great diversity in the types of water bodies, including fast‐flowing streams, ephemeral and permanent water bodies (ponds), even inundated burrows or water‐holding plants, and the position in the water column where they reside, that is the surface, the bottom, or in the open water (Duellman & Trueb, 1986). Of the 178 exotrophic tadpoles, we sampled there were 136 lentic species (inhabit still fresh water), and 37 lotic species (inhabit rapidly moving fresh water), and 5 that were classified as both if they have been found in flowing streams as well as still ponds. Guilds falling under these two divisions ten and eight, respectively) may be unique or may have the same name and definition. For example, guilds defined by the position spent feeding in the water (“benthic’ if they are bottom‐dwellers, and ‘nektonic’ if they swim freely in the water—often in a vertical position), are found in both lentic and lotic systems. However, guilds such as the lotic‐adherent behaviors (clasping, adherent, and suctorial), are only found in lotic systems. Altig and McDiarmid (1999b) provide a summary classification of Anura families and genera by ecomorphological guild. In our Australian species, we have four lentic guilds: 54 obligate benthic species, 28 obligate nektonic species, 53 that are observed to be both (free range though the water body), and a single species that performs vermiform burrowing, using a spiral motion to burrow through thick algal mats into peat substrate in deep peat swamp ponds (*Spicospina flammocaerulea, *fossorial). In the lotic system, there were a total of nine obligate benthic species, and 28 species that have specialized in adhering to rocks with their oral disc in fast‐flowing water. There were also five species that are benthic dwellers but are known from both lentic and lotic systems. It is clear, there is a lot of diversity in tadpole ecology and Anstis” descriptions suggest that guilds are not mutually exclusive; tadpoles can belong to more than one.

**Figure 1:**
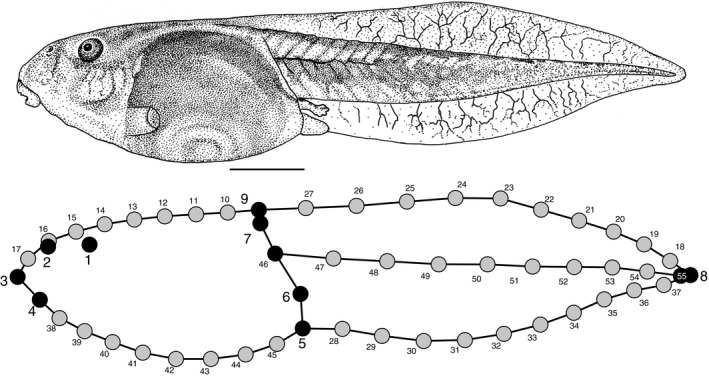
A digitized tadpole **(** *Litoria dahlii* for example purposes**) **with 9 landmarks and 46 semilandmarks. Scale bar is 5 mm. Drawing reproduced with permission from Anstis 2003. Landmarks (black) and semilandmarks (gray) defined as: 1, center of the eye; 2, center of the external nares; 3, point where the upper labium contacts the head/body in lateral view; 4, point where the lower labium contacts the head/body in lateral view; 5, intersection of the head/body and tail on the ventral side, anterior to the vent; 6, intersection of the ventral edge if the tail muscle and the head/body; 7, intersection of the dorsal edge if the tail muscle and the head/body; 8, tip of the tail; 9, point on the dorsal fin closest to landmark 7 (superficially denotes the intersection of the head/body and tail regions); 10–17, equally‐spaced semilandmarks marking the curve of the dorsal aspect of the head/body when viewed laterally; 18–27, equally‐spaced semilandmarks marking the curve of the dorsal aspect of the tail fin when viewed laterally; 28–37, equally‐spaced semilandmarks marking the curve of the ventral aspect of the tail fin when viewed laterally; 38–45, equally‐spaced semilandmarks marking the curve of the ventral aspect of the head/body when viewed laterally; 46–55, equally‐spaced semilandmarks marking the curve of the notochord. Landmark configuration has been straightened from the eye to the tip of the tail along the notochord in order to standardize all specimens’ configurations (see Methods for details)

We characterized tadpole body shape in 2‐dimensions (2D) from left‐lateral view drawings using a geometric morphometric approach, digitizing landmarks and semilandmarks to capture the lateral profile (Figure 1), details of which are in Sherratt et al. (2017). In summary, the drawings were digitised using tpsDig2 v.2.26 (Rohlf, 2016) and routines written in the R statistical environment v.3.3.3 (R Development Core Team, 2017) and ImageJ (Schneider, Rasband, & Eliceiri, 2012). The landmark configurations were straightened using “unbend specimens” function of tpsUtil v.1.74 (Rohlf, 2015); the eye, notochord semilandmarks and tip of tail were used to straighten and standardize the configuration, removing the shape differences due to position of the tail relative to the head/body. The landmark and semilandmark coordinates were then aligned using a generalized Procrustes superimposition (Rohlf & Slice, 1990) implemented in *geomorph* (Adams, Collyer, Kaliontzopoulou, & Sherratt, 2016), where the semilandmarks were permitted to slide along their tangent directions in order to minimize bending energy (Gunz, Mitterocker, & Bookstein, 2005). The resulting Procrustes shape coordinates were subjected to principal component analysis (PCA) to visualize the body shape variation among species in a low‐dimensional morphospace. These procedures and the following analyses were performed in the R statistical environment v.3.3.3 (R Development Core Team, 2017) unless otherwise stated.

Morphological disparity of the ecomorphological guilds, which is the amount of variation in body shape in morphospace, was measured using the Procrustes variance (Zelditch, Swiderski, & Sheets, 2012). To test whether there are statistical differences in mean body shape between the ecomorphological guilds and assess how distinct they are from each other, we first used a permutational ANOVA for highly‐multidimensional data, with post‐hoc pairwise tests for differences between each guild. Given the pattern may be driven by phylogenetic relatedness, we also used a phylogenetic ANOVA (pANOVA), which involves a phylogenetic generalized least squares analysis for multivariate data (Adams, 2014), again with post‐hoc pairwise tests. Both tests were implemented in *geomorph* (Adams et al., 2016). We used a published phylogenetic hypothesis from a previous study of Australian tadpoles (Sherratt et al., 2017) which is a Bayesian molecular phylogeny with branch lengths for 166 species. For the pANOVA, the dataset was subsampled to include only these species for which there is phylogenetic information. We also performed a pANOVA on the exotrophic species only to test the effect of ecology (water source) and behavior (foraging position in water), to assess which has a greater effect on the observed variation of tadpole body shapes.

To compare our 187 sampled Australian species to the gamut ecomorphological guilds of larval anurans described by Altig and Johnston (1989), we digitized (as above) the tadpole drawings by Linda Trueb in *Biology of Amphibians* (Duellman & Trueb, 1986), which were reprinted and provided as examples for the ecomorphological guilds in Altig and Johnston (1989) and later in Altig and McDiarmid (1999b). We digitized 17 of the 18 species presented, which are all exotrophic and predominantly New World (2 Asian and 1 African). We excluded the arboreal bufonid *Mertensophryne anotis* (previously *Stephopaedes anotis*) because the unique crown around the head obscures the eyes and nostrils in the lateral view. Species names were updated herein based on those published by AmphibiaWeb (online database). The landmark configurations of the 204 species were aligned by generalized Procrustes superimposition and subjected to a PCA as above to visualize the morphospace.

## 3 RESULTS

**Figure 2:**
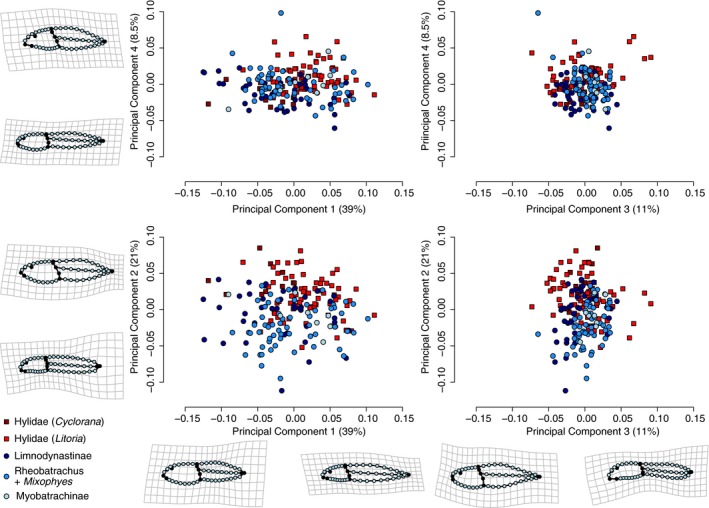
Morphospace of Australian tadpoles. A principal component (PC) analysis of 188 species reveals four axes describing 79.5% of the total variance. Scatter plots for PCs 1 to 4 are shown, along with thin‐plate splines representing shape changes from the mean shape (grid) to the minima and maxima of each axis. Point shapes in the scatterplots represent family designation, and colors for main clades within each family

Principal component analysis (PCA) of tadpole body shape for the 188 Australian species recovered four PCs describing a total of 79.5% of the total variance (Figure 2). The remaining PCs each describe less that 5% of the shape variance and are not discussed further. Shape changes described by these four PC axes are explained below using the terminology of Anstis (2013), and illustrated in Figure 2. PC1 (39.1%) describes the relative length of the tail; shape changes from the sample mean in the negative direction along PC1 relate to a shortening of the tail to be equal lengths with the head/body, and toward the positive direction the tail lengthens to be more than double the length of the head/body. Change in oral disc position along this axis is from anteroventral to near‐ventral (negative to positive). PC2 (20.9%) describes dorsoventral compression of the whole tadpole; shape changes from the mean in the negative direction relate to a flat‐head/body and tail, and toward the positive end the tail is expanded with highly arched fins and the head/body is rounded. Oral disc position also changes along this axis from ventral to anteroventral (negative to positive). PC3 (10.9%) describes tail‐tip shape variation; shape changes from the mean in the negative direction relate to moderately arched fins and a pointed tail with slight dorsal flexion, and toward the positive end the tail is blunt and ventrally flexed. Change in oral disc position along this axis is from anterior (negative) to near‐ventral (positive). PC4 (8.55%) describes head/body and tail shape, particularly relating to the dorsal fin shape; shape changes from the mean in the negative direction relate to a long, paddle‐shaped tail, that is dorsoventrally deeper posteriorly than medially because the dorsal fin is moderately arched posteriorly; toward the positive end the tail is shorter and fins highly arched ending in a narrow point. Oral disc position also changes along this axis from near‐ventral to anteroventral (negative to positive).

Species are distributed in morphospace with some phylogenetic structure: hylids and myobatrachids (Limnodynastinae, *Rheobatrachus*, *Mixophes* and Myobatrachinae) share in most of the observed variety of tadpole body shapes, although the two families do not fully overlap in morphospace (Figure 2) indicating there are aspects of the morphology unique to each family. For example, many hylids (*Litoria* species) occupy the most extreme region of morphospace defining the highly arched tail fins and finely pointed tail tip, which are characteristic of nektonic behavior. The most extremely situated myobatrachids, those with deep bodies and short tails (negative end of PC1), comprise mostly species that are found in very arid areas, and are predominantly burrowing as adults.

**Figure 3:**
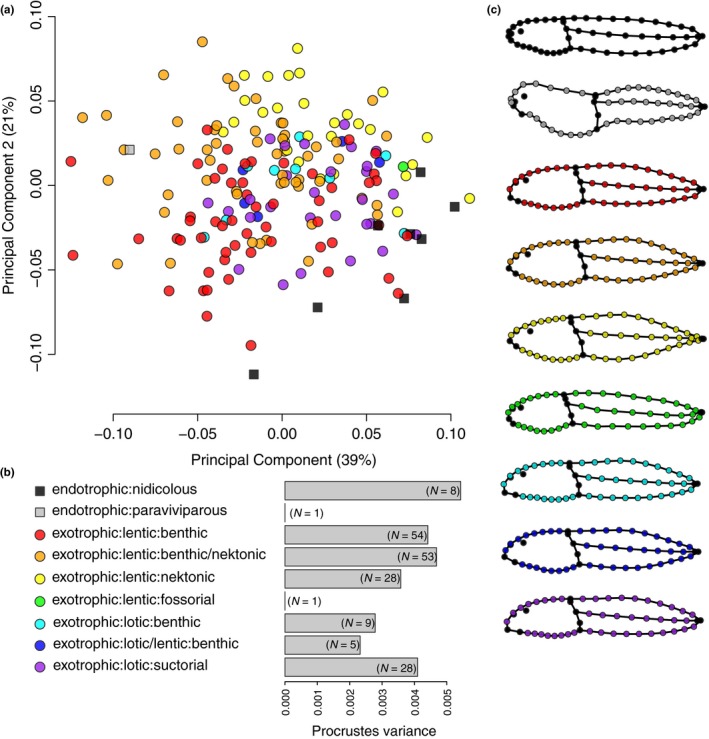
Ecomorphological guilds of Australian tadpoles in morphospace. (a) Scatter plot of the first two PC axes (60% of the total variance; see Figure 2) with guilds mapped onto species points by color. (b) morphological disparity for each guild, with number of species (N). (c) average body shapes of the ecomorphological guilds represented as consensus landmark configurations (or the actual landmark configuration when *N* = 1), all at the same scale

There is structure to the Australian tadpole body shape morphospace relating the ecomorphology (Figure 3). Guilds partition the morphospace with a lot of overlap (Figure 3a) and there is substantial within‐guild variation in body shape (Figure 3b). Guilds are represented by mostly distinct average body shapes (Figure 3c). A standard ANOVA suggests there are distinct and significant differences among guilds for mean body shape (*R* ^2^ = 0.238, *F*_(6,178)_ = 9.2812, *p* = 0.001). However, in a phylogenetic context, there is no statistical difference between guilds (pANOVA, *R* ^2^ = 0.037, *F*_(6,157)_ = 1.009, *p* = 0.407; only guilds with more than three species compared, Table 1). Mean body shapes for the guilds of Australian tadpoles are shown in Figure 3c. Endotrophic tadpoles have a clearly distinct body shape from exotrophic species and are situated at the periphery of the tadpole morphospace, yet they are still a part of the continuum of observed body shape variation. The body shape of the endotrophic‐paraviviparous stomach‐developing *R. silus* (gray square, Figure 3a), is clearly very different from the endotrophic‐nidicolous species (terrestrial‐nest developers, black squares, Figure 3a), which occupy a narrow and elongate region of space at the positive end of PC1. Along this is subtle shape variation around an elongate tadpole shape, with a small head/body and long tail that has shallow fins, either ending bluntly or tapering off.

**Table 1:** Results of a standard ANOVA and a phylogenetic ANOVA (pANOVA) to test for differences in body shape between guilds

ANOVA	Endotrophic: nidicolous	Exotrophic: lentic: benthic	Exotrophic: lentic: bent/nekt	Exotrophic: lentic: nektonic	Exotrophic: lotic: benthic	Exotrophic: lotic: suctorial	Exotrophic: lotic/lentic: benthic
Endotrophic: nidicolous	‐	0.001	**0.001**	**0.001**	**0.002**	**0.002**	**0.012**
Exotrophic: lentic: benthic	0.087	‐	**0.001**	**0.001**	0.055	**0.001**	0.239
Exotrophic: lentic: bent/nekt	0.104	0.037	‐	**0.001**	**0.03**	**0.001**	0.353
Exotrophic: lentic:nektonic	0.096	0.075	0.053	‐	**0.003**	**0.001**	0.1
Exotrophic: lotic: benthic	0.081	0.041	0.045	0.062	‐	0.818	0.894
Exotrophic: lotic: suctorial	0.072	0.049	0.059	0.068	0.020	‐	0.553
Exotrophic: lotic/lentic: benthic	0.079	0.041	0.037	0.049	0.026	0.032	‐
Number of species	8	54	53	28	9	5	28

pANOVA	Endotrophic:nidicolous	Exotrophic: lentic: benthic	Exotrophic: lentic: bent/nekt	Exotrophic: lentic: nektonic	Exotrophic: lotic: benthic	Exotrophic: lotic: suctorial	Exotrophic: lotic/lentic: benthic
Endotrophic: nidicolous	‐	0.591	0.254	0.673	0.64	0.559	0.99
Exotrophic: lentic: benthic	0.069	‐	0.892	0.646	0.976	0.918	0.959
Exotrophic: lentic: bent/nekt	0.095	0.037	‐	0.09	0.321	0.111	0.572
Exotrophic: lentic:nektonic	0.070	0.052	0.055	‐	0.126	0.045	0.911
Exotrophic: lotic: benthic	0.072	0.033	0.049	0.060	‐	0.706	0.809
Exotrophic: lotic: suctorial	0.072	0.033	0.051	0.062	0.026	‐	0.859
Exotrophic: lotic/lentic: benthic	0.047	0.048	0.067	0.043	0.053	0.048	‐
Number of species	8	50	48	25	6	3	24

Note

Pairwise differences are given as Procrustes distances between least squares (LS) means for the groups (lower triangle), and associated P‐values in the upper triangle, based on 1,000 permutations. Bold *p*‐values indicate significance at the 5% level (*α* = 0.05). The number of species in each guild is given above. See Figure 3 for additional guild analyses.

**Figure 4:**
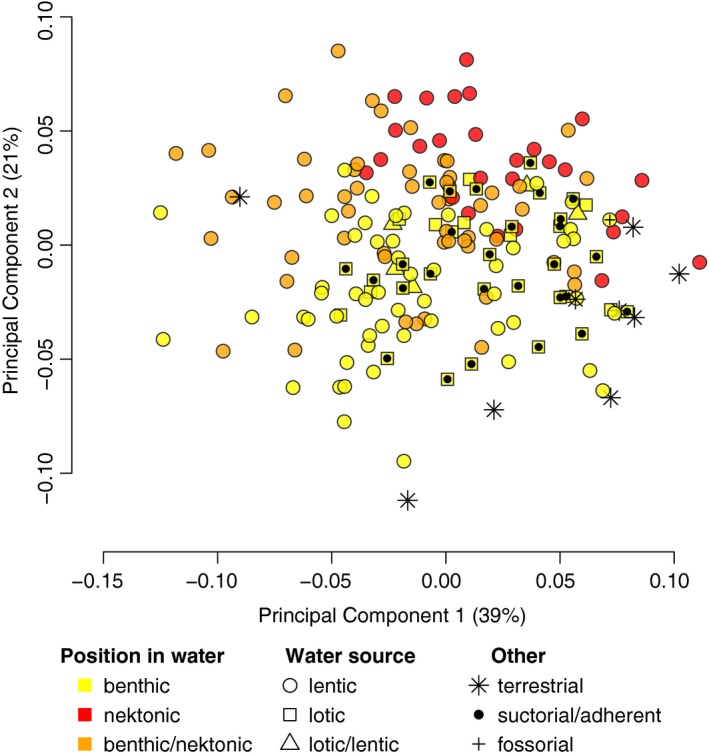
The diversity of Australian tadpole ecology in morphospace. Scatter plot of the first two PC axes (60% of the total variance; see Figure 2) with ecological and behavioral classification mapped onto species points. Exotrophic tadpoles, those that develop and feed in an aquatic environment, are marked by point shape indicating those that occur in freely‐moving water (“lotic,” square) and still water (“lentic,” circle), or both (triangle). These tadpoles can also swim freely in open water (“nektonic,” red), be a bottom‐dweller (“benthic,” yellow), or display both behaviors (orange). Overlaid point shape are exotrophic species that are also specialized in adhering to rocks under water (•), or perform vermiform burrowing (+). Endotrophic taxa, that develop in a terrestrial environment, are also shown (*)

Simplifying the classification of tadpoles by broader ecological and behavioral categories provides a clearer picture of the variation in Australian tadpoles (Figure 4). Yet there is no significant effect of ecology (the type of water source) on exotrophic tadpole body shape (pANOVA, *R* ^2^ = 0.015, *F*_(2,152)_ = 1.17, *p* = 0.259), or in foraging position in water—a behavioral trait (*R* ^2^ = 0.014, *F*_(3,152)_ = 1.09, *p* = 0.329). Effect sizes (F scores) are adjusted for nested effects. Most species sampled in this study are lentic (circles, Figure 4), these are distributed over the whole morphospace indicating a great range of morphologies. Lotic species are fewer, and occupy a small area of morphospace at the positive end of PC1 and the negative end of PC2 (squares, Figure 4). However, the position in the water clearly partitions the species in the morphospace. Interestingly, species that display both benthic/nektonic behaviors, or are found in lotic and lentic water sources, appear intermediate in tadpole morphospace.

**Figure 5:**
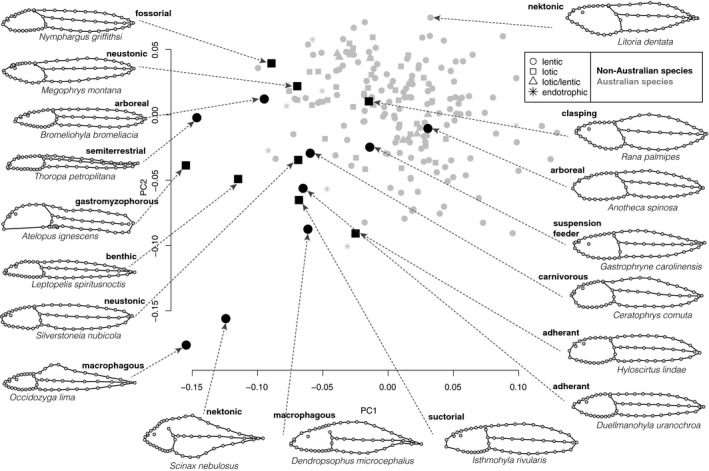
Ecomorphological guilds of tadpoles in morphospace. Scatter plot of the first two axes of a PCA (35.8% and 22.9% of the total shape variation) including endotrophic and exotrophic Australian species (from Anstis, 2013) and exotrophic non‐Australian species (from Altig & Johnston, 1989). PC1 here is reversed compared with Figures 2, 3, and 4. Landmark configurations are arranged around the morphospace to show the shape diversity. *Atelopus ignescens* and *Megophrys montana* are outliers at the positive and negative ends of PC3, respectively (13.6%, not shown), while the remaining species all cluster together at the middle of PC3. Terminology definitions not provided in Methods: *gastromyzophorous*—has an abdominal sucker which extends from the lip past the middle of the abdomen; *neustonic*—surface feeder; *macrophagous*—feed by taking larger bites (compared to the smaller particles of rasping tadpoles); *carnivorous*—feed on macroinvertebrates, and conspecific and heterospecific tadpoles; *suspension feeder*—harvests suspended particles by pumping water through the oral disc; *arboreal*—live and feed in isolated water‐filled cavities, elevated or not (adapted from Lannoo, Townsend, & Wassersug, 1987)

Australia's diversity of tadpoles occupies only a small proportion of the variation described by the ecomorph examples from Altig and Johnston (1989) from a worldwide sample (Figure 5). Morphological disparity of the non‐Australian species (Procrustes variance = 0.016) is more than twice that of Australian taxa (0.006). Australian and non‐Australian species are partially overlapping, indicating novel morphologies to each sample.

## 4 DISCUSSION

The external morphology of larval frogs has long been thought to be an adaptation to different ecological conditions or ways of life (Altig & Johnston, 1989; Altig & McDiarmid, 1999b; Orton, 1953). Since these seminal papers, it has been shown that tadpoles inhabiting specific types of water bodies or have particular feeding strategies tend to display similar body shapes (e.g., van Buskirk, 2009, Baldo et al., 2014, Haad, Vera Candioti, & Baldo, 2011), thus supporting the guild framework. Here, we used the classification of ecomorphological guilds proposed by Altig and Johnston (1989) to examine the extent to which body shape relates to specific ecomorphological guilds, the amount of variability within each guild, and the degree of continuity in shape among guilds, at a continental scale. Our results suggest that there is a lot of morphological variability within and amongst guilds that cannot be differentiated from similarity by ancestry alone, and that Australia's ecomorphological diversity is limited with respect to worldwide species.

Guilds of Australian tadpoles exhibited visibly different body shapes, but we found substantial within‐guild variation and overlap among the categories and the mean guild body shapes were not significantly different after accounting for phylogeny. A similarly large‐scale study (Marques & Nomura, 2015) of morphological diversity across guilds for 101 species of anurans from Central and South America revealed comparable results to ours: that there is great morphological diversity within guilds, and substantial overlap of guilds in morphospace. Their results revealed significant differences in tadpole body shape among guilds, but they did not take into account phylogenetic relatedness in their model. Unfortunately, they did not describe the morphological changes associated with the morphospace axes or specific guilds to provide comparison with our study. Microhabitat use in tadpoles is known to be plastic and can differ substantially among tadpoles of closely‐related species (Eterovick et al., 2010). Therefore, the observed morphological diversity in our study and that of Marques and Nomura (2015) is likely an indication that morphological‐specificity is not strictly necessary for many microhabitats and tadpoles are potentially more generalist and adaptable than we expect.

Tadpole body shape diversity among species is heritable and arises early in development (e.g., Strauss & Altig, 1992), but ecological factors are known to promote morphological plasticity. For example, different water environments (flowing streams and still ponds) and dietary conditions induce repeatable variation in tadpole body size and shape (Doughty & Roberts, 2003; Jennings & Scott, 1993). Also, the presence of their kin can induce morphogenesis from one ecomorphological phenotype to another (e.g., omnivore to carnivore, Pfennig & Frankino, 1997, Frankino & Pfennig, 2001). Finally, higher temperatures are known to induce morphological changes resulting in relatively larger head/bodies (Merilä & Björklund, 1999). Therefore while there is support for an adaptive basis to the body shape of at least some tadpoles and a relationship between morphology and locomotive performance, the influence of environmental plasticity cannot be ignored.

Classifying species by behavior and ecological microhabitats (i.e., Figure 3) rather than using guilds (i.e., Figure 4) is potentially a more relevant descriptor of the morphological variation observed. The sampled exotrophic tadpoles showed a morphological continuum between lentic and lotic species, with those that are known to inhabit both types of water systems lying intermediate in morphospace (Figure 4). Our results, comprising three families over the Australian continent, support what has been found at a smaller scale in two intrageneric studies of ecomorphological tadpole body shape within a single genus of South American bufonid tadpoles (Baldo et al., 2014; Haad et al., 2011); the authors recovered a morphological continuum in morphospace with similar morphological changes associated with lotic and lentic species as shown here (i.e., degree of tail fin arching and total elongation). Thus, there is evidence of similar morphological response to similar ecological pressures, and warrants further investigation into convergent evolution in a phylogenetic framework. It is evident that some taxa are more generalist and capable of surviving in rapidly moving or still fresh water (lotic/lentic respectively). Future work to characterize the microenvironment inhabited by tadpoles and their adults in more detail, such as quantifying water depth and currents (e.g., Eterovick et al., 2010) may better explain the continuum of body shape variation observed.

In Figure 5, we bring the observed diversity of Australian tadpoles into a global context. Visually comparing the main axes of Australian tadpole body shape (thin‐plate splines, Figure 2) and the distribution of Australian species in a morphospace containing other species (Figure 5) indicates that Australia's tadpole diversity represents a small portion of documented tadpole diversity in the ecomorphological guilds of Altig and Johnston (1989). Many of the guilds they described do not exist in Australia's fauna. The region of morphospace occupied only by the Australian species we sampled (positive values on PC1 and 2) describes species with relatively large head/body regions compared to tail. However, it is clear from the dense‐sampling of this study that the Australian exotrophic tadpoles are more homogenous, representing subtle variants on the same theme of a couple of guilds. Australia's tadpole fauna lacks many of the Altig and Johnston (1989) guilds, in particular lacking any of the morphologically unique “extreme tadpoles,” such as the elongate fossorial *Leptobrachella mjobergi* (Haas, Hertwig, & Das, 2006), or the head “crown” adorned arboreal bufonids (Channing, 1978; Müller, Measey, & Malonza, 2005). Therefore, we conclude that the ecomorphological variation of Australia's tadpoles is limited compared to what we know of worldwide diversity. We encourage further study of the limiting factors in ecological niche diversity that may be governing the tadpole assemblages of Australia.

We recognize that not all ecomorphological guilds are expected to share similar external body shapes as described here, because they may relate to particular dietary or feeding specializations that only pertain to specific morphological features. Certainly, the external body shape is not the only indicator of ecological specialization in tadpoles; Vera Candioti (2006), Vera Candioti (2007) examined the relationship between hyobranchial skeleton anatomy and ecology in exotrophic‐lentic tadpoles and defined four groups based on the shape of the keratinized oral disc and diet: microphagous, generalized, microphagous and megalophagous. Using a subsample of the species we have studied here, van Buskirk (2009) suggested that there is an adaptive component to tadpole oral disc morphology, as inferred from the relationship between oral disc shape and habitat, which specifically explained differences between still pond (lentic) and fast‐slowing stream (lotic) species. This finding was driven by the morphospace position of the adherent/suctorial species that have very different‐shaped oral discs compared to other species rather than a selection signal from the water system per se. A quantitative approach that takes into account multiple aspects of the tadpole's morphology is thus preferable to better understand the apparent ecomorphological variation. The study by Roelants et al. (2011) is the only globally‐comprehensive analysis of tadpole morphological diversity and is based upon a comprehensive cladistic character dataset of numerous larval traits coded by Haas (2003). They showed that there are four distinct clusters of species in the morphospace corresponding to the four morphotypes of Orton (1953), which were defined based on the keratinized oral disc. In fact, most of the Australian tadpoles studied here belong to Orton's morphotype IV, which is the largest cluster occupied by most anuran taxa of the world. Whether this relates to the limited ecomorphological variation we observed in Figure 5 remains to be tested.

## AUTHORS CONTRIBUTION

ES and JSK designed the study. ES and MA collected the data, ES analysed the data, and ES wrote the manuscript with contributions from MA and JSK. All authors read and approved the final manuscript.

## DATA ACCESSIBILITY

The digitizing protocol (including R computer code) is provided in the Supplementary Methods of Sherratt et al., 2017. Phylogenetic tree are available on Dryad (http://dx.doi.org/10.5061/dryad.23j6t). All raw shape coordinate data is available on Figshare (https://doi.org/10.25909/5be500cf5f0c4).
