# Supplementary material for: Ecomorphological diversity of Australian tadpoles
Source: Ecol Evol. 2018 Nov 26;8(24):12929–39. doi: 10.1002/ece3.4733 (PMC6308865; doi:10.1002/ece3.4733)
Supplement: Supplementary file 1 [file ECE3-8-12929-s001.docx]

**Table S1 List of the 188-sampled species and their ecomorphological guild**

| Species | Taxonomic group | Guilds |
| --- | --- | --- |
| *Adelotus brevis* | Limnodynastinae | exotrophic-lentic-benthic/nektonic |
| *Crinia bilingua* | Myobatrachinae | exotrophic-lentic-benthic |
| *Crinia deserticola* | Myobatrachinae | exotrophic-lentic-benthic |
| *Crinia fimbriata* | Myobatrachinae | exotrophic-lentic-benthic |
| *Crinia flindersensis* | Myobatrachinae | exotrophic-lentic-lotic/adherent |
| *Crinia georgiana* | Myobatrachinae | exotrophic-lentic-benthic |
| *Crinia glauerti* | Myobatrachinae | exotrophic-lentic-benthic |
| *Crinia insignifera* | Myobatrachinae | exotrophic-lentic-benthic |
| *Crinia parinsignifera* | Myobatrachinae | exotrophic-lentic-benthic |
| *Crinia pseudinsignifera* | Myobatrachinae | exotrophic-lentic-benthic |
| *Crinia remota* | Myobatrachinae | exotrophic-lentic-benthic |
| *Crinia riparia* | Myobatrachinae | exotrophic-lentic-lotic/suctorial |
| *Crinia signifera* | Myobatrachinae | exotrophic-lentic-benthic |
| *Crinia sloanei* | Myobatrachinae | exotrophic-lentic-benthic |
| *Crinia subinsignifera* | Myobatrachinae | exotrophic-lentic-benthic |
| *Crinia tasmaniensis* | Myobatrachinae | exotrophic-lentic-benthic |
| *Crinia tinnula* | Myobatrachinae | exotrophic-lentic-benthic |
| *Cyclorana alboguttata* | Hylidae- *Cyclorana* | exotrophic-lentic-benthic/nektonic |
| *Cyclorana australis* | Hylidae- *Cyclorana* | exotrophic-lentic-benthic/nektonic |
| *Cyclorana brevipes* | Hylidae- *Cyclorana* | exotrophic-lentic-benthic/nektonic |
| *Cyclorana cultripes* | Hylidae- *Cyclorana* | exotrophic-lentic-benthic/nektonic |
| *Cyclorana longipes* | Hylidae- *Cyclorana* | exotrophic-lentic-benthic/nektonic |
| *Cyclorana maculosa* | Hylidae- *Cyclorana* | exotrophic-lentic-benthic/nektonic |
| *Cyclorana maini* | Hylidae- *Cyclorana* | exotrophic-lentic-benthic/nektonic |
| *Cyclorana manya* | Hylidae- *Cyclorana* | exotrophic-lentic-benthic/nektonic |
| *Cyclorana novaehollandiae* | Hylidae- *Cyclorana* | exotrophic-lentic-benthic/nektonic |
| *Cyclorana platycephala* | Hylidae- *Cyclorana* | exotrophic-lentic-benthic/nektonic |
| *Cyclorana vagita* | Hylidae- *Cyclorana* | exotrophic-lentic-benthic/nektonic |
| *Cyclorana verrucosa* | Hylidae- *Cyclorana* | exotrophic-lentic-benthic/nektonic |
| *Geocrinia alba* | Myobatrachinae | endotrophic-nidicolous |
| *Geocrinia laevis* | Myobatrachinae | exotrophic-lentic-benthic |
| *Geocrinia leai* | Myobatrachinae | exotrophic-lentic-benthic |
| *Geocrinia lutea* | Myobatrachinae | endotrophic-nidicolous |
| *Geocrinia rosea* | Myobatrachinae | endotrophic-nidicolous |
| *Geocrinia victoriana* | Myobatrachinae | exotrophic-lentic-benthic |
| *Geocrinia vitellina* | Myobatrachinae | endotrophic-nidicolous |
| *Heleioporus albopunctatus* | Limnodynastinae | exotrophic-lentic-benthic/nektonic |
| *Heleioporus australiacus* | Limnodynastinae | exotrophic-lentic-benthic/nektonic |
| *Heleioporus barycragus* | Limnodynastinae | exotrophic-lentic-benthic/nektonic |
| *Heleioporus eyrei* | Limnodynastinae | exotrophic-lentic-benthic/nektonic |
| *Heleioporus inornatus* | Limnodynastinae | exotrophic-lentic-benthic/nektonic |
| *Heleioporus psammophilus* | Limnodynastinae | exotrophic-lentic-benthic/nektonic |
| *Lechriodus fletcheri* | Limnodynastinae | exotrophic-lentic-benthic |
| *Limnodynastes convexiusculus* | Limnodynastinae | exotrophic-lentic-benthic/nektonic |
| *Limnodynastes depressus* | Limnodynastinae | exotrophic-lentic-benthic/nektonic |
| *Limnodynastes dorsalis* | Limnodynastinae | exotrophic-lentic-benthic/nektonic |
| *Limnodynastes dumerili* | Limnodynastinae | exotrophic-lentic-benthic/nektonic |
| *Limnodynastes dumerilii fryi* | Limnodynastinae | exotrophic-lentic-benthic/nektonic |
| *Limnodynastes dumerilii grayi* | Limnodynastinae | exotrophic-lentic-benthic/nektonic |
| *Limnodynastes dumerilii insularis* | Limnodynastinae | exotrophic-lentic-benthic/nektonic |
| *Limnodynastes dumerilii variegata* | Limnodynastinae | exotrophic-lentic-benthic/nektonic |
| *Limnodynastes fletcheri* | Limnodynastinae | exotrophic-lentic-benthic/nektonic |
| *Limnodynastes interioris* | Limnodynastinae | exotrophic-lentic-benthic/nektonic |
| *Limnodynastes lignarius* | Limnodynastinae | exotrophic-lentic-lotic/suctorial |
| *Limnodynastes peronii* | Limnodynastinae | exotrophic-lentic-benthic/nektonic |
| *Limnodynastes salmini* | Limnodynastinae | exotrophic-lentic-benthic/nektonic |
| *Limnodynastes tasmaniensis* | Limnodynastinae | exotrophic-lentic-benthic/nektonic |
| *Limnodynastes terraereginae* | Limnodynastinae | exotrophic-lentic-benthic/nektonic |
| *Litoria adelaidensis* | Hylidae-*Litoria* | exotrophic-lentic-nektonic |
| *Litoria aurea* | Hylidae-*Litoria* | exotrophic-lentic-nektonic |
| *Litoria aurifera* | Hylidae-*Litoria* | exotrophic-lotic-benthic |
| *Litoria barringtonensis* | Hylidae-*Litoria* | exotrophic-lotic-benthic |
| *Litoria bicolor* | Hylidae-*Litoria* | exotrophic-lentic-nektonic |
| *Litoria booroolongensis* | Hylidae-*Litoria* | exotrophic-lotic-benthic |
| *Litoria brevipalmata* | Hylidae-*Litoria* | exotrophic-lentic-nektonic |
| *Litoria burrowsae* | Hylidae-*Litoria* | exotrophic-lentic-nektonic |
| *Litoria caerulea* | Hylidae-*Litoria* | exotrophic-lentic-nektonic |
| *Litoria castanea* | Hylidae-*Litoria* | exotrophic-lentic-nektonic |
| *Litoria cavernicola* | Hylidae-*Litoria* | exotrophic-lentic-benthic/nektonic |
| *Litoria chloris* | Hylidae-*Litoria* | exotrophic-lotic/lentic-benthic |
| *Litoria citropa* | Hylidae-*Litoria* | exotrophic-lotic-benthic/suctorial |
| *Litoria cooloolensis* | Hylidae-*Litoria* | exotrophic-lentic-nektonic |
| *Litoria coplandi* | Hylidae-*Litoria* | exotrophic-lotic-benthic/suctorial |
| *Litoria cyclorhyncha* | Hylidae-*Litoria* | exotrophic-lentic-nektonic |
| *Litoria dahlii* | Hylidae-*Litoria* | exotrophic-lentic-benthic/nektonic |
| *Litoria daviesae* | Hylidae-*Litoria* | exotrophic-lotic-benthic/suctorial |
| *Litoria dentata* | Hylidae-*Litoria* | exotrophic-lentic-nektonic |
| *Litoria electrica* | Hylidae-*Litoria* | exotrophic-lentic-nektonic |
| *Litoria eucnemis* | Hylidae-*Litoria* | exotrophic-lotic-benthic |
| *Litoria ewingii* | Hylidae-*Litoria* | exotrophic-lentic-nektonic |
| *Litoria freycineti* | Hylidae-*Litoria* | exotrophic-lentic-benthic/nektonic |
| *Litoria gilleni* | Hylidae-*Litoria* | exotrophic-lentic-nektonic |
| *Litoria gracilenta* | Hylidae-*Litoria* | Exotrophic-lotic/lentic-benthic |
| *Litoria inermis* | Hylidae-*Litoria* | exotrophic-lentic-benthic/nektonic |
| *Litoria infrafrenata* | Hylidae-*Litoria* | exotrophic-lentic-nektonic |
| *Litoria jervisiensis* | Hylidae-*Litoria* | exotrophic-lentic-nektonic |
| *Litoria jungguy* | Hylidae-*Litoria* | exotrophic-lotic-benthic/suctorial |
| *Litoria latopalmata* | Hylidae-*Litoria* | exotrophic-lentic-benthic/nektonic |
| *Litoria lesueurii* | Hylidae-*Litoria* | exotrophic-lotic-benthic/suctorial |
| *Litoria littlejohni* | Hylidae-*Litoria* | exotrophic-lentic-nektonic |
| *Litoria longirostris* | Hylidae-*Litoria* | exotrophic-lotic-benthic |
| *Litoria lorica* | Hylidae-*Litoria* | exotrophic-lotic-benthic/suctorial |
| *Litoria meiriana* | Hylidae-*Litoria* | exotrophic-lotic-benthic |
| *Litoria microbelos* | Hylidae-*Litoria* | exotrophic-lentic-benthic/nektonic |
| *Litoria moorei* | Hylidae-*Litoria* | exotrophic-lentic-nektonic |
| *Litoria myola* | Hylidae-*Litoria* | exotrophic-lotic/lentic-benthic |
| *Litoria nannotis* | Hylidae-*Litoria* | exotrophic-lotic-benthic/suctorial |
| *Litoria nasuta* | Hylidae-*Litoria* | exotrophic-lentic-benthic/nektonic |
| *Litoria nigrofrenata* | Hylidae-*Litoria* | exotrophic-lentic-benthic/nektonic |
| *Litoria nudidigita* | Hylidae-*Litoria* | exotrophic-lotic-benthic |
| *Litoria nyakalensis* | Hylidae-*Litoria* | exotrophic-lotic/suctorial |
| *Litoria olongburensis* | Hylidae-*Litoria* | exotrophic-lentic-nektonic |
| *Litoria pallida* | Hylidae-*Litoria* | exotrophic-lentic-benthic/nektonic |
| *Litoria paraewingi* | Hylidae-*Litoria* | exotrophic-lentic-nektonic |
| *Litoria pearsoniana* | Hylidae-*Litoria* | exotrophic-lotic-benthic |
| *Litoria peronii* | Hylidae-*Litoria* | exotrophic-lentic-nektonic |
| *Litoria personata* | Hylidae-*Litoria* | exotrophic-lotic-lentic-nektonic/suctorial |
| *Litoria phyllochroa* | Hylidae-*Litoria* | exotrophic-lotic-benthic |
| *Litoria raniformis* | Hylidae-*Litoria* | exotrophic-lentic-nektonic |
| *Litoria revelata* | Hylidae-*Litoria* | exotrophic-lentic-nektonic |
| *Litoria rheocola* | Hylidae-*Litoria* | exotrophic-lotic/suctorial |
| *Litoria rothii* | Hylidae-*Litoria* | exotrophic-lentic-nektonic |
| *Litoria rubella* | Hylidae-*Litoria* | exotrophic-lentic-nektonic |
| *Litoria serrata* | Hylidae-*Litoria* | exotrophic-lotic/lentic-benthic |
| *Litoria spenceri* | Hylidae-*Litoria* | exotrophic-lotic-benthic |
| *Litoria splendida* | Hylidae-*Litoria* | exotrophic-lentic-nektonic |
| *Litoria staccato* | Hylidae-*Litoria* | exotrophic-lotic-suctorial |
| *Litoria subglandulosa* | Hylidae-*Litoria* | exotrophic-lotic-benthic/suctorial |
| *Litoria tornieri* | Hylidae-*Litoria* | exotrophic-lentic-benthic/nektonic |
| *Litoria tyleri* | Hylidae-*Litoria* | exotrophic-lentic-nektonic |
| *Litoria verreauxii* | Hylidae-*Litoria* | exotrophic-lentic-nektonic |
| *Litoria verreauxii alpina* | Hylidae-*Litoria* | exotrophic-lentic-nektonic |
| *Litoria watjulumensis* | Hylidae-*Litoria* | exotrophic-lentic-lotic-benthic/nektonic |
| *Litoria wilcoxii* | Hylidae-*Litoria* | exotrophic-lotic-benthic/suctorial |
| *Litoria xanthomera* | Hylidae-*Litoria* | exotrophic-lotic/lentic-benthic |
| *Mixophyes balbus* | *Rheobatrachus* & *Mixophyes* | exotrophic-lotic-benthic/suctorial |
| *Mixophyes carbinensis* | *Rheobatrachus* & *Mixophyes* | exotrophic- lotic-benthic/suctorial |
| *Mixophyes coggeri* | *Rheobatrachus* & *Mixophyes* | exotrophic- lotic-benthic/suctorial |
| *Mixophyes fasciolatus* | *Rheobatrachus* & *Mixophyes* | exotrophic-lotic-lentic-benthic/suctorial |
| *Mixophyes fleayi* | *Rheobatrachus* & *Mixophyes* | exotrophic-lotic-benthic/suctorial |
| *Mixophyes iteratus* | *Rheobatrachus* & *Mixophyes* | exotrophic-lotic-lentic-benthic/suctorial |
| *Mixophyes schevilli* | *Rheobatrachus* & *Mixophyes* | exotrophic-lotic-benthic/suctorial |
| *Neobatrachus albipes* | Limnodynastinae | exotrophic-lentic-benthic/nektonic |
| *Neobatrachus aquilonius* | Limnodynastinae | exotrophic-lentic-benthic/nektonic |
| *Neobatrachus kunapalari* | Limnodynastinae | exotrophic-lentic-benthic/nektonic |
| *Neobatrachus pelobatoides* | Limnodynastinae | exotrophic-lentic-benthic/nektonic |
| *Neobatrachus pictus* | Limnodynastinae | exotrophic-lentic-benthic/nektonic |
| *Neobatrachus sudellae* | Limnodynastinae | exotrophic-lentic-benthic/nektonic |
| *Neobatrachus sutor* | Limnodynastinae | exotrophic-lentic-benthic/nektonic |
| *Neobatrachus wilsmorei* | Limnodynastinae | exotrophic-lentic-benthic/nektonic |
| *Notaden bennetti* | Limnodynastinae | exotrophic-lentic-benthic |
| *Notaden melanoscaphus* | Limnodynastinae | exotrophic-lentic-benthic |
| *Notaden nichollsi* | Limnodynastinae | exotrophic-lentic-benthic |
| *Notaden weigeli* | Limnodynastinae | exotrophic-lentic-benthic |
| *Papurana daemeli* | Ranidae | exotrophic-lotic-lentic-benthic |
| *Paracrinia haswelli* | Myobatrachinae | exotrophic-lentic-benthic/nektonic |
| *Philoria frosti* | Limnodynastinae | endotrophic-nidicolous |
| *Philoria kundagungan* | Limnodynastinae | endotrophic-nidicolous |
| *Philoria loveridgei* | Limnodynastinae | endotrophic-nidicolous |
| *Philoria sphagnicolus* | Limnodynastinae | endotrophic-nidicolous |
| *Platyplectrum ornatum* | Limnodynastinae | exotrophic-lentic-benthic |
| *Platyplectrum spenceri* | Limnodynastinae | exotrophic-lentic-benthic |
| *Pseudophryne australis* | Myobatrachinae | exotrophic-lentic-benthic |
| *Pseudophryne bibroni* | Myobatrachinae | exotrophic-lentic-benthic |
| *Pseudophryne coriacea* | Myobatrachinae | exotrophic-lentic-benthic |
| *Pseudophryne corroboree* | Myobatrachinae | exotrophic-lentic-benthic |
| *Pseudophryne covacevichae* | Myobatrachinae | exotrophic-lentic-benthic |
| *Pseudophryne dendyi* | Myobatrachinae | exotrophic-lentic-benthic |
| *Pseudophryne guentheri* | Myobatrachinae | exotrophic-lentic-benthic |
| *Pseudophryne major* | Myobatrachinae | exotrophic-lentic-benthic |
| *Pseudophryne occidentalis* | Myobatrachinae | exotrophic-lentic-benthic |
| *Pseudophryne pengilleyi* | Myobatrachinae | exotrophic-lentic-benthic |
| *Pseudophryne raveni* | Myobatrachinae | exotrophic-lentic-benthic |
| *Pseudophryne semimarmorata* | Myobatrachinae | exotrophic-lentic-benthic |
| *Rheobatrachus silus* | *Rheobatrachus* & *Mixophyes* | endotrophic-paraviviparous |
| *Spicospina flammocaerulea* | Myobatrachinae | exotrophic-lentic-fossorial |
| *Taudactylus acutirostris* | Myobatrachinae | exotrophic-lotic-adherent |
| *Taudactylus diurnus* | Myobatrachinae | exotrophic-lotic-adherent |
| *Taudactylus eungellensis* | Myobatrachinae | exotrophic-lotic-adherent |
| *Taudactylus liemi* | Myobatrachinae | exotrophic-lotic-adherent |
| *Uperoleia altissima* | Myobatrachinae | exotrophic-lentic-benthic |
| *Uperoleia arenicola* | Myobatrachinae | exotrophic-lentic-benthic |
| *Uperoleia aspera* | Myobatrachinae | exotrophic-lentic-benthic |
| *Uperoleia borealis* | Myobatrachinae | exotrophic-lentic-benthic |
| *Uperoleia crassa* | Myobatrachinae | exotrophic-lentic-benthic |
| *Uperoleia daviesae* | Myobatrachinae | exotrophic-lentic-benthic |
| *Uperoleia fusca* | Myobatrachinae | exotrophic-lentic-benthic |
| *Uperoleia glandulosa* | Myobatrachinae | exotrophic-lentic-benthic |
| *Uperoleia inundata* | Myobatrachinae | exotrophic-lentic-benthic |
| *Uperoleia laevigata* | Myobatrachinae | exotrophic-lentic-benthic |
| *Uperoleia lithomoda* | Myobatrachinae | exotrophic-lentic-benthic |
| *Uperoleia littlejohni* | Myobatrachinae | exotrophic-lentic-benthic |
| *Uperoleia martini* | Myobatrachinae | exotrophic-lentic-benthic |
| *Uperoleia mimula* | Myobatrachinae | exotrophic-lentic-benthic |
| *Uperoleia mjobergi* | Myobatrachinae | exotrophic-lentic-benthic |
| *Uperoleia rugosa* | Myobatrachinae | exotrophic-lentic-benthic |
| *Uperoleia talpa* | Myobatrachinae | exotrophic-lentic-benthic |
| *Uperoleia tyleri* | Myobatrachinae | exotrophic-lentic-benthic |
